# Editorial: Effective Strategies to Develop Rural Health Workforce in Low and Middle-Income Countries (LMICs)

**DOI:** 10.3389/fpubh.2021.702362

**Published:** 2021-06-04

**Authors:** Belinda Gabrielle O'Sullivan, Ian Couper, Pratyush Kumar, Matthew Richard McGrail

**Affiliations:** ^1^Rural Clinical School, University of Queensland, Toowoomba, QLD, Australia; ^2^Ukwanda Centre for Rural Health, Stellenbosch University, Cape Town, South Africa; ^3^Department of Family Medicine, Sir Ganga Ram Hospital, New Delhi, India

**Keywords:** LMICs, rural workforce, training, education, recruitment, retention, rural pathways, grow your own

Around the world, rural communities are facing similar challenges related to accessing the healthcare that they need. These challenges are most noticeable in low- and middle-income countries (LMICs) where the highest proportion of people lives in rural and remote under-resourced communities. The best way to access affordable care in these areas is to focus on building enough skilled health workers in these communities. This relies on LMICs rural communities adopting “grow your own” approaches, involving training local health workers to the scope of skills needed in the community and fostering their connection to the community through support, mentoring and good working conditions (O'Sullivan et al.). The steps involved in “grow your own” strategies are called “rural pathways” and the World Health Organization recommends that rural pathways are critical as part of a suite of strategies to improve health workforce retention (1, 2). For rural LMICs, investing in rural-based training for health workers, adds to the progress toward health, social, and economic progress in line with the Sustainable Development Goals (3). This is because this investment can dramatically lift local educational opportunities and increase access to jobs in locations where they are most needed (4).

However, despite the rich evidence from high income countries, there is a shortage of published evidence about rural pathways strategies in LMICs settings (1, 2). This special call was an attempt to foster exemplars of these, as a means of promoting cross-learning and reflection about the unique LMICs rural context. By sharing information about rural pathways, it is possible for effective approaches in one community to be adopted and scaled up elsewhere, for improving rural workforce capacity.

With this background in mind, this special call brings together a collection of examples of education and training strategies, tools, and resources, from LIMICs of different world regions. We are pleased to have attracted a diverse representation of rural pathways strategies fit to a wide range of LMICs rural contexts as we know that every region has unique experiences to share.

The first article describes a WHO sponsored rural pathways checklist designed to assist LMICs to apply evidence-informed approaches to plan and benchmark rural pathways to achieve community health goals (O'Sullivan et al.). The checklist includes eight holistic steps involved in rural pathways action and a range of prompts to encourage LMICs stakeholders to consider the potential of people already in rural and remote communities to be trained in health roles in their community, considerations in fostering local training, and ensuring the right professional support to retain health staff (O'Sullivan et al.).

Fonken et al. extended on this work, by applying the checklist to review Kyrgyzstan's current pathway to develop the rural primary care workforce and prioritize the next steps. Using the checklist prompts, his team identified shared goals for strengthening recruitment by addressing working conditions and improving the efficiency of clinics.

Amin et al. further drew from the rural pathways philosophy to evaluate nurses' performance for their central role in delivering primary healthcare in rural tribal areas of Rajasthan, India. The research identified that most nurses were sourced from rural and tribal communities that the clinics serve; nurses from these communities were likely to have a higher retention than those from urban areas. Sourcing from rural and tribal communities, providing on-going training in clinical and social skills, and using a non-hierarchical work environment, coupled with individualized mentoring assisted with health worker motivation.

In the Asia Pacific region, Putri et al. did a pivotal national cross-sectional survey about how to foster early career doctors to work more remotely. Dr. Putri's work provides objective evidence that selecting doctors with a remote background, giving them remote work experience and providing financial incentives, can increase the uptake of work in remote Indonesian areas.

The Philippines has a strong track record in “grow your own” rural workforce training strategies (5, 6) and a new study led by Guignona et al. describes the degree to which a rural education program has impacted on local rural health services and communities in this country. Research about Training for Health Equity Network (THEnet) programs (7), also showed that training that is structured to deliver on the needs of the community produces a health workforce with strong intent to practice rurally and in generalist or primary care disciplines (Johnston et al.).

Several other case studies included in this special topic, delve into how to deliver training in the LMICs context, including in areas like the Pacific Islands and Lesotho (Bryden et al. and McIver et al.). McIvor et al. describe the establishment of new postgraduate training programmes in family, community, and rural hospital medicine in Timor-Leste, Tonga, Solomon Islands, and Vanuatu which have been crucial in building a health workforce in the Pacific region of an adequate size, distribution, and skill level necessary to meet the needs of rural communities across the “Blue Continent” (McIver et al.). Bryden et al., describe the development of the first family medicine speciality training program in the small mountainous kingdom of Lesotho, in southern Africa, which is the first accredited postgraduate medical training program in that country (Bryden et al.).

Larkins et al. describes real-world training in public health investigations, targeted at rural/provincial and regional health and biosecurity workers and managers from Fiji, Indonesia, Papua New Guinea (PNG), Solomon Islands, and Timor-Leste. By mentoring and teaching skills to investigate real-world problems, over 50 projects were done by health workers aiding the analysis and mapping of surveillance data, infection control, and outbreak management. This is incredibly valuable for preparing rural LMICs for local pandemic responses.

Rural pathways are often complemented by regulatory strategies to enhance workforce supply. Mahat et al. discuss strategies to overcome the critical shortage of rural physicians in Nepal using a national medical scholarship program targeting rural public hospital service of 2 years. Of 1,226 scholarships only 12–57% of the first 4 cohorts completed their service, increasing to 86–98% in the most recent three cohorts. Overall, 78% of service days were completed in rural hospitals.

Across other health disciplines, Müller and Couper evaluated the success of the Collaborative Care Project (CCP), which exposes rural clinical school participants in one South African training program, to interprofessional home visits (Müller and Couper). They found students had increased understanding of how a team approach to primary health care contributes to improved patient care, job satisfaction, better discharge planning and continuity of care. In particular, students gained understanding of these issues within low resourced rural contexts and also what limitations the context places on ideal patient care, with issues of power and hierarchy being critical in this.

Finally, rural pathways only work if rural workers are supported once they have been trained. Wheatley et al. describes supporting doctors in isolated rural postings, *via* the Rural Family Medicine Café (RFMC) social media project. The project effectively engaged and supported those interested in rural family medicine thus decreasing occupational isolation.

Overall, the range of high-quality contributions to this special call provides impetus to continue the process of fostering LMICs rural pathways evidence and we commend Frontiers for leading such opportunities. We are also grateful for the ongoing support and encouragement of the WONCA working party on rural practice (rural WONCA) and similar agencies who continue to promote the agenda of global equity of access to healthcare.

## Author Contributions

All authors listed have made a substantial, direct and intellectual contribution to the work, and approved it for publication.

## Conflict of Interest

The authors declare that the research was conducted in the absence of any commercial or financial relationships that could be construed as a potential conflict of interest.
